# Hepatoprotective Effects of Bioflavonoid Luteolin Using Self-Nanoemulsifying Drug Delivery System

**DOI:** 10.3390/molecules26247497

**Published:** 2021-12-11

**Authors:** Faiyaz Shakeel, Moad M. Alamer, Prawez Alam, Abdullah Alshetaili, Nazrul Haq, Fars K. Alanazi, Sultan Alshehri, Mohammed M. Ghoneim, Ibrahim A. Alsarra

**Affiliations:** 1 Kayyali Chair for Pharmaceutical Industries, Department of Pharmaceutics, College of Pharmacy, King Saud University, Riyadh 11451, Saudi Arabia; m.m.alamer@hotmail.com (M.M.A.); nazrulhaq59@gmail.com (N.H.); afars@ksu.edu.sa (F.K.A.); 2 Department of Pharmacognosy, College of Pharmacy, Prince Sattam Bin Abdulaziz University, Al-Kharj 11942, Saudi Arabia; prawez_pharma@yahoo.com; 3 Department of Pharmaceutics, College of Pharmacy, Prince Sattam Bin Abdulaziz University, Al-Kharj 11942, Saudi Arabia; a.alshetaili@psau.edu.sa; 4 Department of Pharmaceutics, College of Pharmacy, King Saud University, Riyadh 11451, Saudi Arabia; salshehri1@ksu.edu.sa (S.A.); ialsarra@ksu.edu.sa (I.A.A.); 5 Department of Pharmacy Practice, College of Pharmacy, AlMaarefa University, Ad Diriyah 13713, Saudi Arabia; mghoneim@mcst.edu.sa

**Keywords:** bioflavonoid, droplet size, hepatoprotective effects, luteolin, SNEDDS

## Abstract

Luteolin (LUT) is a natural pharmaceutical compound that is weakly water soluble and has low bioavailability when taken orally. As a result, the goal of this research was to create self-nanoemulsifying drug delivery systems (SNEDDS) for LUT in an attempt to improve its in vitro dissolution and hepatoprotective effects, resulting in increased oral bioavailability. Using the aqueous phase titration approach and the creation of pseudo-ternary phase diagrams with Capryol-PGMC (oil phase), Tween-80 (surfactant), and Transcutol-HP (co-emulsifier), various SNEDDS of LUT were generated. SNEDDS were assessed for droplet size, polydispersity index (PDI), zeta potential (ZP), refractive index (RI), and percent of transmittance (percent T) after undergoing several thermodynamic stability and self-nanoemulsification experiments. When compared to LUT suspension, the developed SNEDDS revealed considerable LUT release from all SNEDDS. Droplet size was 40 nm, PDI was <0.3, ZP was −30.58 mV, RI was 1.40, percent T was >98 percent, and drug release profile was >96 percent in optimized SNEDDS of LUT. For in vivo hepatoprotective testing in rats, optimized SNEDDS was chosen. When compared to LUT suspension, hepatoprotective tests showed that optimized LUT SNEDDS had a substantial hepatoprotective impact. The findings of this investigation suggested that SNEDDS could improve bioflavonoid LUT dissolution rate and therapeutic efficacy.

## 1. Introduction

The chemical name of luteolin (LUT) is 2-(3,4-dihydroxyphenyl)-5,7-dihydroxy-4*H*-chromen-4-one [1,2]. Celery, perilla leaf, chamomile tea, and green pepper all contain this poorly soluble bioactive flavonoid [3,4]. It has antioxidant [5], anti-inflammatory [6,7,8], anti-allergic [6], anti-amnestic [9], hepatoprotective [10], cardioprotective [3], neuroprotective [8,9], and anticancer [11,12,13,14] properties. Although it has been shown to be a good bioactive chemical for treating liver problems, due to its limited solubility and bioavailability after oral administration, substantial doses are necessary [10].

Various formulation approaches, including complexation with cyclodextrin [15,16], complexation with phospholipid [10,17], complexation with cyclosophoraoses [18], cocrystal technology [19], palmitoylethanolamide/LUT composite [20], and microparticles [21,22], were investigated to modify its physicochemical properties, which would finally results in enhancement in solubility, dissolution rate, therapeutic activity, and bioavailability. The solubility of LUT in water was reported to be 1.0 mg/mL at ambient temperature [4,23]. Because LUT has a low aqueous solubility, it has a low in vitro dissolution rate, which means it has a low oral bioavailability [23]. 

The development of nanocarrier-based drug delivery systems for bioactive compounds/nutraceuticals in order to improve bioavailability and therapeutic efficacy while minimizing side effects has sparked a great deal of attention recently [24,25,26,27]. SNEDDS can encapsulate hydrophobic bioactive compounds/nutraceuticals into their internal oil phase, boosting medication solubility, therapeutic efficacy, and bioavailability, and minimizing side effects [26,27]. SNEDDS can generate very tiny nanoemulsions (less than 100 nm in size) when diluted with an aqueous media such as gastrointestinal (GI) fluids or water [28,29,30]. SNEDDS have been utilized for a long time to increase the solubility, GI permeability, bioavailability, and therapeutic effects of a number of poorly soluble bioactive natural compounds [29,30,31,32,33,34,35,36,37]. LUT has recently been explored using nanotechnology-based drug carriers such as copolymer micelles [38], solid-lipid nanoparticles [39], zein-based nanoparticles [40], and liposomes [41] to improve its bioavailability and bioactivity in animal models. The antioxidant and anti-inflammatory potential of LUT SNEDDS has also been studied [29]. Despite this, the hepatoprotective effects of LUT when it is encapsulated in SNEDDS have not been studied. As a result, these studies were conducted in order to develop multiple SNEDDS formulations of LUT using pseudo-ternary phase diagrams and low energy emulsification techniques in order to increase its hepatoprotective properties. Capryol-PGMC (oil phase), Tween-80 (surfactant), Transcutol-HP (co-emulsifier), and water (aqueous phase) were used to create different SNEDDS formulations of LUT. 

## 2. Results and Discussion

### 2.1. Equilibrium Solubility Data of LUT in Different Components

The major criterion for component screens were the equilibrium solubility data of LUT in different components [42,43]. Table 1 shows the equilibrium solubility values of LUT in various components at 25 °C. Among the several oil phases tested, Capryol-PGMC had the highest solubility of LUT (25.72 ± 1.74 mg/g), followed by Capryol-90, Lauroglycol-90, Lauroglycol-FCC, Triacetin, and sesame oil. Among the several surfactants studied, Tween-80 (18.52 ± 0.81 mg/g) had the highest solubility of LUT, followed by Cremophor-EL and Labrasol. Transcutol-HP, on the other hand, had the highest solubility of LUT (68.32 ± 2.83 mg/g), followed by isopropanol (IPA), ethanol, propylene glycol (PG), and ethylene glycol (EG), among the several co-emulsifiers studied. Equilibrium solubility of LUT in water was found to be 0.03 ± 0.00 mg/g. Equilibrium solubility of LUT in water at 37 °C was estimated as 50.60 µg/mL by Qing et al. (2017) [38]. The solubility of LUT as mole fraction in water at 25 °C was recorded as 1.75 × 10^−6^ (converted as 27.80 µg/g) elsewhere [23]. Equilibrium solubility of LUT in water at 25 °C was recorded as 30 µg/g in the present work, which was very close to the literature values. The solubility of LUT as a mole fraction in ethanol and IPA at 25 °C has been reported as 1.85 × 10^−3^ (converted as 11.50 µg/g) and 1.94 × 10^−3^ (converted as 9.25 mg/g), respectively, by Peng et al. (2006) [3]. The solubility of LUT as mole fraction in ethanol, IPA, EG, PG, and Transcutol-HP at 25 °C has been reported as 1.88 × 10^−3^ (converted as 11.70 mg/g), 2.51 × 10^−3^ (converted as 12.00 mg/g), 1.30 × 10^−3^ (converted as 6.00 mg/g), 2.12 × 10^−3^ (converted as 8.00 mg/g), and 3.09 × 10^−2^ (converted as 68.00 mg/g), respectively, by Shakeel et al. (2018) [23]. Equilibrium solubility of LUT in ethanol, IPA, EG, PG, and Transcutol-HP was obtained as 11.84 mg/g, 12.13 mg/g, 6.07 mg/g, 8.24 mg/g, and 68.32 mg/g, respectively, in the preset research work, which were also very close to the literature values [3,23]. Based on the equilibrium solubility data of LUT, Capryol-PGMC (oil phase), Tween-80 (surfactant), and Transcutol-HP (co-emulsifier) were selected as the optimum components for the preparation LUT SNEDDS. Water was selected as the aqueous phase due to its “availability, cost effectiveness, and frequent use” in the literature [42,43,44]. 

### 2.2. Construction of Pseudo-Ternary Phase Diagrams for the Preparation of LUT SNEDDS 

**Figure 1:**
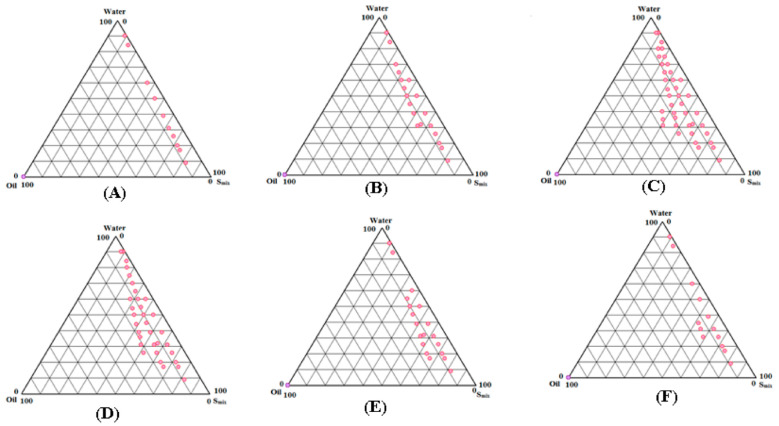
Pseudo-ternary phase diagrams for the preparation of the SNEDDS zones of LUT for oil phase (Capryol-PGMC), surfactant (Tween-80), co-emulsifier (Transcutol-HP), and aqueous phase (water) at S_mix_ ratios of (**A**) 1:0, (**B**) 1:2, (**C**) 1:1, (**D**) 2:1, (**E**) 3:1, and (**F**) 4:1.

The “low energy emulsification technique” was used to create different SNEDDS formulations of LUT by creating pseudo-ternary phase diagrams with Capryol-PGMC (oil), Tween-80 (surfactant), Transcutol-HP (co-emulsifiers), and water (aqueous phase) [43,44]. Figure 1 depicts phase diagrams for various surfactant to co-emulsifier (S_mix_) ratios. 

When compared to the other S_mix_ ratios tested, the 1:0 S_mix_ ratio (Figure 1A) showed the lowest SNEDDS zones. However, when compared to S_mix_ ratio of 1:0, the S_mix_ ratio of 1:2 (Figure 1B) revealed slightly greater SNEDDS zones. In contrast to the other S_mix_ ratios studied, the 1:1 S_mix_ ratio (Figure 1C) produced the highest SNEDDS zones. When the S_mix_ ratio of 2:1 (Figure 1D) was investigated, the SNEDDS zones began to shrink once more. The SNEDDS zones of 2:1 S_mix_ ratio were slightly lower than 1:1 ratio but higher than the other S_mix_ ratios studied. The SNEDDS zones of S_mix_ ratio of 3:1 (Figure 1E) and 4:1 (Figure 1F) were further decreased compared with S_mix_ ratios of 1:1 and 2:1. S_mix_ ratios of 1:1 were used to indicate the maximal SNEDDS zones (Figure 1C). As a result, different SNEDDS formulations for LUT were chosen from Figure 1C. The whole SNEDDS zones in Figure 1C were taken into consideration for formulation selection, keeping in mind the solubilization of the oil phase (Capryol-PGMC) with respect to S_mix_. From Figure 1C, varied concentrations of Capryol-PGMC (12, 16, 20, 24, and 28 percent *w/w*) were combined with constant amounts of Tween-80 (20 percent *w/w*) and Transcutol-HP (20 percent *w/w*) to make SNEDDS. Each SNEDDS included 20 mg of LUT, and the resulting formulations were labeled LSN1-LSN5. Table 2 shows the LSN1-LSN5 chemical compositions.

### 2.3. Thermodynamic Stability Tests 

For the elimination of unstable or metastable formulations during the low energy emulsification method, various thermodynamic tests were performed. Centrifugation, heating and cooling cycles, and freeze-pump-thaw cycles were all used in these studies [28,29,35]. Table 3 shows the qualitative findings of these tests. After centrifugation, heating and cooling cycles, and freeze-pump-thaw cycles, all of the SNEDDS formulations were confirmed to be stable. As a result, these formulations were chosen for self-nanoemulsification testing.

### 2.4. Self-Nanoemulsification Tests 

The goal of this experiment was to see if there was any phase separation or precipitation with three different diluents: water, 0.1 N HCl, and phosphate buffer (pH 6.8) [35]. Table 3 displays the qualitative outcomes of this examination. In the presence of all three diluents, the results revealed that all LUT SNEDDS (LSN1-LSN5) passed this test with grade A. Furthermore, there was no evidence of LUT precipitation during a self-nanoemulsification test in the presence of water, 0.1 N HCl, or phosphate buffer (pH 6.8), implying that LUT in the form of SNEDDS was stable under aqueous, stomach, and intestinal pH conditions. 

### 2.5. Physicochemical Characterization of SNEDDS

In order to ensure the proper formation of LUT SNEDDS in nanosized range, prepared LUT SNEDDS were characterized physicochemically. Table 4 displays the results for these parameters. The droplet size (Z-average) of various LUT SNEDDS (LSN1-LSN5) was recorded as 48.58–124.58 nm using a Malvern Zetasizer. The Z-average value of SNEDDS was found to be enhanced significantly with an increase in the amount of Capryol-PGMC/oil phase (*p* < 0.05). The Z-average value was inversely proportional with the amount of Capryol-PGMC in the formulations. The maximum Z-average value was recorded in formulation LSN5 (124.58 ± 9.41 nm). This result was most likely attributable to the existence of the highest concentration of Capryol-PGMC (28.0 percent *w/w*) in LSN5. The lowest Z-average value (48.58 ± 2.47 nm) was attained in formulation LSN1. LSN1 had the lowest Z-average value, which was most likely owing to the existence of the lowest concentration of Capryol-PGMC (12.0 percent *w/w*). The impact of S_mix_ concentrations was not studied in this work. It has been frequently reported in the literature that the Z-average value of SNEDDS/nanoemulsions is increased with increases in the concentration of the oil phase in the formulation [42,43]. Our findings were consistent with those previously published in the literature.

The polydispersity indices (PDIs) of various LUT SNEDDS (LSN1-LSN5) were obtained in the range of 0.168–0.293. The lower PDI values for all formulations showed droplet homogeneity. The lowest PDI was obtained for formulation LSN1, indicating the most uniform size distribution. However, the formulation LSN5 yielded the highest PDI (0.293).

The zeta potential (ZP) values for various LUT SNEDDS (LSN1-LSN5) range from −23.74 to −30.58 mV. These values were not substantially different amongst SNEDDS (*p* > 0.05). The stability of prepared SNEDDS was shown by ZP values in the magnitude of ±30.0 mV [28,35]. 

For various LUT SNEDDS (LSN1-LSN5), the refractive indices (RIs) were recorded as 1.344–1.349. The RIs of various SNEDDS were not substantially different (*p* > 0.05). The recorded RIs for all SNEDDS were very near to the RI of water (RI = 1.33), showing that various LUT SNEDDS have a “transparent nature and oil-water (*o/w*) type behavior” [28]. 

For various LUT SNEDDS (LSN1-LSN5), the turbidity/percent of transmittance (percent T) values were recorded as 94.27–98.94 percent (Table 4). Formulation LSN1 yielded the highest percent T (98.94 ± 0.53 percent). Formulation LSN5, on the other hand, had the lowest percent T (94.27 ± 1.09 percent). The highest and lowest percent T of formulations LSNI and LSN5 were possible due to the lowest and highest droplet size of formulations LSN1 and LSN5, respectively. The “optical clarity and translucent nature” of the prepared SNEDDS was demonstrated by the greater percent T values in all SNEDDS.

**Figure 2:**
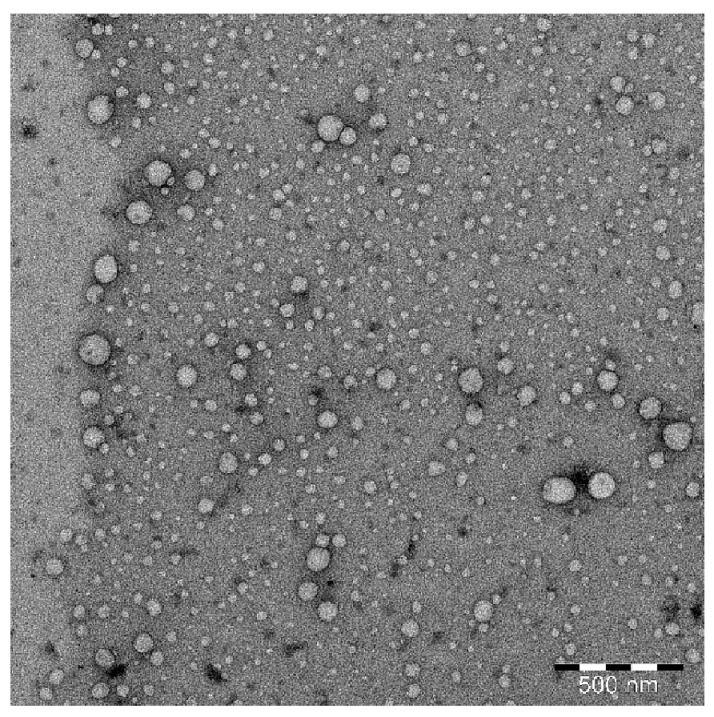
Transmission electron microscopy (TEM) image of optimized LUT-SNEDDS (LSN1) showing spherical-shaped droplets within nanometer range.

The surface texture/morphology and size distribution of an optimized SNEDDS LSN1 were studied using transmission electron microscopy (TEM). Figure 2 shows a TEM picture of the SNEDDS LSN1. The droplets of SNEDDS LSN1 were spherical and scattered within a nanometer range. The presence of Tween-80 and Transcutol-HP was most likely responsible for the droplets’ spherical shape. 

### 2.6. In Vitro Drug Release Studies

**Figure 3:**
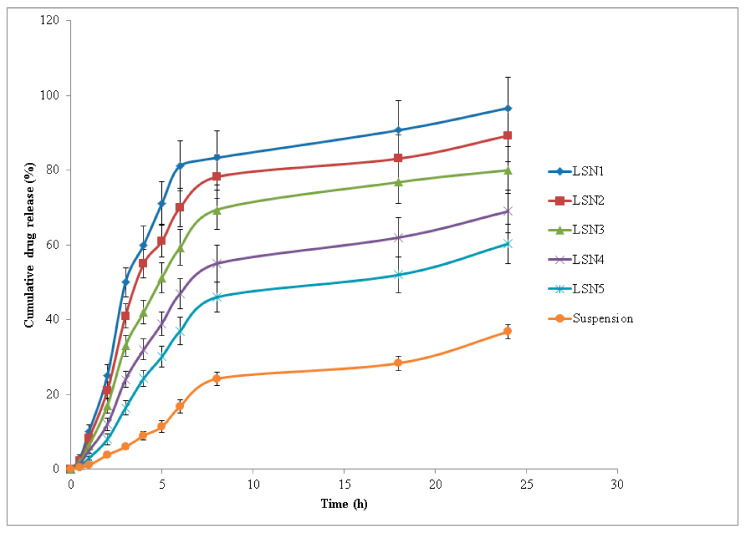
In vitro drug release profile of LUT via dialysis bag (mean ± SD, *n* = 3) from various SNEDDS (LSN1-LSN5) and aqueous suspension of LUT.

In vitro drug release tests were performed to assess the release profile of LUT from LUT SNEDDS (LSN1-LSN5) and LUT suspension via “Dialysis Bag” in order to find the best formulation. Figure 3 shows the results of LUT release from various SNEDDS (LSN1-LSN5) and LUT suspension. The initial release of LUT from all SNEDDS and LUT suspension was observed as rapid (i.e., immediate drug release profile). When compared to LUT suspension, the rate of LUT release from all SNEDDS (LSN1-LSN5) was greater (*p* < 0.05). For up to 6 h, the immediate release profile of LUT from all SNEDDS and LUT suspension were recorded (Figure 3). After 6 h, all SNEDDS and LUT suspensions showed slow LUT release (i.e., sustained release drug profile). The highest release of LUT was seen in the SNEDDS formulation LSN1 (Figure 3). After 24 h of investigation, the cumulative percent release of LUT from LSN1 was 96.6 percent, compared to 36.8 percent from LUT suspension. Within 6 h of the trial, more than 81 percent of LUT was released from LSN1. In LUT suspension, the minimal drug release profile was observed. The cumulative percent release of LUT increased considerably with the reduction in droplet size of the formulation among distinct SNEDDS (LSN1-LSN5) (*p* < 0.05). The highest release profile of LUT from formulation LSN1 was the most likely because of its small droplet size and the presence of the minimum amount of Capryol-PGMC. The presence of the minimum amount of oil phase, i.e., Capryol-PGMC, would result in reduction in droplet size. This results in increased surface area for the release of LUT in the dissolution media [28]. The release profile of LUT from different SNEDDS (LSN1-LSN5) and LUT suspension in two steps (i.e., immediate release profile in first step and sustained release profile in second step) suggested the “diffusion controlled dissolution rate” of LUT [28,43].

### 2.7. Drug Release Kinetics

Various parameters of release kinetics for LUT SNEDDS (LSN1-LSN5) and LUT aqueous suspension were obtained using various computational models such as zero order, first order, Higuchi, Hixon-Crowell, and the Korsemeyer–Peppas model [45,46]. Table 5 displays the results of this analysis. Based on the values of correlation coefficients (R^2^) obtained for different models, the release pattern of LUT from formulations LSN1-LSN5 and LUT suspension followed the Korsemeyer–Peppas model because the R^2^ values were recorded as maximum for this model. The mechanism of drug release was evaluated based the recorded values of diffusion coefficients (n). Based on n values, formulations LSN1-LSN4 followed the Korsemeyer–Peppas model with non-Fickian diffusion mechanism because the value of n was less than 1.0 for these formulations. However, the formulation LSN5 and LUT suspension followed the Korsemeyer–Peppas model with supercase II transport mechanism because the n value was greater than 1.0 for LSN5 and LUT suspension (Table 5).

If the value of n = 0.5, this suggests the Fickian diffusion mechanism. However, if n > 0.5 but n < 1.0, it suggests the non-Ficknian diffusion mechanism. On the other hand, if n > 1.0, it suggests the supercase II transport mechanism [45,46]. The n value in formulations LSN1–LSN4 was obtained as 0.978–0.972, suggesting a non-Ficknian diffusion mechanism. However, the n value for formulation LSN5 and LUT suspension was obtained as 1.201 and 1.368, respectively, suggesting the supercase II transport mechanism. In the literature, many hydrophobic compounds followed the Korsemeyer–Peppas model with non-Fickian diffusion mechanism from SNEDDS or oral nanoemulsions [43,47,48]. Therefore, the results of drug release kinetics of LUT from most of the studied SNEDDS were in good agreement with those reported in the literature.

### 2.8. Hepatoprotective Effects

Formulation LSN1 was chosen as an optimized SNEDDS of LUT for further hepatoprotective study based on maximum drug release (96.6 percent), minimum Z-average value (48.58 nm), and the presence of a minimum concentration of Capryol-PGMC. Table 6 displays the results of hepatoprotective evaluation. The animals in the control group (Group I) had normal levels of serum alanine aminotransferase (ALT), serum aspartate aminotransferase (AST), γ-glutamyl transpeptidase (γ-GGT), serum bilirubin, and serum alkaline phosphatase (ALP), among other group biochemical markers. Oral CCl_4_ dosing (Group II animals) resulted in a substantial increase in AST, ALT, ALP, γ-GGT, and bilirubin (*p* < 0.01). Standard (i.e., silymarin; Group III), LUT suspension (Group IV), and optimized LUT-SNEDDS LSN1 (Group V) oral delivery resulted in substantial reductions in AST, ALT, ALP, γ-GGT, and bilirubin levels compared to the toxic control (Group II animals) (*p* < 0.01). 

Table 7 shows the findings of the hepatoprotective evaluation of liver tissue. It was discovered that the animals in the control group had normal levels of catalase (CAT), glutathione peroxidase (GSH), superoxide dismutase (SOD), and melondialdehyde (MDA) in their tissues. GSH levels in the control group were assessed to be 1.19 ± 0.03 nmol/mg. In Group II rats, however, oral treatment with CCl_4_ lowered the GSH value to 0.45 ± 0.01 nmol/mg. Furthermore, when compared to the control group, silymarin suspension, LUT suspension, and SNEDDS LSN1 treatment decreased GSH levels (Table 7). The concentrations of CAT, MDA, and SOD in liver tissues were calculated as 47.71 ± 1.29 U/mg, 3.25 ± 0.07 nmol/mg, and 24.86 ± 1.34 U/mg, respectively, in the control. In Group II rats, oral treatment with CCl_4_ resulted in substantial reductions in CAT, MDA, and SOD levels in liver tissues (*p* < 0.05). When compared to the control, oral treatment of silymarin suspension, LUT suspension, and SNEDDS LSN1 lowered CAT, MDA, and SOD levels (Table 7). In comparison to control, the hepatoprotective efficacies of silymarin, LUT suspension, and SNEDDS LSN1 were likewise significant (*p* < 0.05). Table 6 summarizes the typical serum values of AST, ALT, ALP, γ-GGT, and bilirubin in rats [49]. Silymarin suspension, LUT suspension, and SNEDDS LSN1 have all been found to be effective in lowering AST, ALT, and ALP levels. However, silymarin was found to be the most effective in reducing the levels of AST, ALT, and ALP. In comparison to the control, oral delivery with CCl_4_ resulted in a significant increase in γ-GGT and bilirubin levels. However, the oral treatment with silymarin, LUT, and SNEDDS LSN1 resulted in a marked reduction in γ-GGT and bilirubin levels compared with Group II rats. 

The liver contains many forms of transaminases, such as serum AST, ALT, and ALP, and their levels in the blood have been seen to increase in individuals with liver diseases [35]. In the examination of hepatocellular injury, serum AST, ALT, and ALP have been identified as specific indicators. The most common method for assessing hepatocellular damage is to measure serum bilirubin and ALP levels [50,51]. The levels of bilirubin, AST, ALT, and ALP in the proposed study were considerably higher than in the control group. Oral dosing of silymarin, LUT, and SNEDSS LSN1 resulted in significant hepatoprotective effects. Table 7 lists the typical amounts of CAT, GSH, MDA, and SOD found in rat liver tissues [49]. GSH was found to be vital in the liver’s cellular activity [35]. It also detoxifies organic chemicals to control gene expression, apoptosis, and cellular transport. Free radicals and other reactive oxygen species are efficiently scavenged by enzymes. GSH is critical for the regular functioning of cells and tissues. Hepatic damage was produced by its significant depletion [35,52,53,54]. In the suggested investigation, GSH depletion was seen after CCl_4_ delivery compared to a control group. Oral treatment of silymarin, LUT, and SNEDDS LSN1 restored hepatotoxicity by raising liver GSH levels. It is well known that CCl_4_’s free radical causes peroxidative breakdown, resulting in MDA formation and membrane damage. MDA levels in the liver are connected to lipid peroxidation, which causes tissue damage and the failure of antioxidant defense mechanisms [55,56,57]. In the planned investigation, MDA levels were drastically lowered after oral treatment with silymarin, LUT, and SNEDDS LSN1. In comparison to hazardous CCl_4_, silymarin, LUT, and SNEDDS LSN1 dramatically boosted CAT and SOD levels following oral treatment. Hepatoprotective effects are aided by elevated SOD and CAT levels. Overall, the findings of this investigation indicated that the developed SNEDDS LSN1 had stronger hepatoprotective effects compared to the toxic control (Group II animals) against CCl_4_-induced liver injury.

## 3. Materials and Methods

### 3.1. Materials

LUT was obtained from Beijing Mesochem Technology Pvt. Ltd. (Beijing, China). Lauroglycol-90, Lauroglycol-FCC, Capryol-90, Capryol-PGMC, Labrasol, and Transcutol-HP were obtained from Gattefosse (Lyon, France). Triacetin was obtained from Alpha Chemica (Mumbai, India). Chromatography grade acetonitrile, Tween-80, and sesame oil were obtained from Sigma-Aldrich (St. Louis, MO, USA). Cremophor-EL was obtained from BASF (Cheshire, UK). EG, PG, ethanol, and IPA were obtained from E-Merck (Darmstadt, Germany). Chromatography grade water was collected from Milli-Q water purification. All other chemicals and reagents used were of analytical/pharmaceutical grades. 

### 3.2. HPLC Method for LUT Estimation

The estimation of LUT in all studied samples was carried out using a validated HPLC method [23]. The chromatographic identification of LUT was achieved at room temperature (25 ± 1 °C) using a Waters HPLC system (Waters, USA) attached to a 1515 isocratic HPLC pump, 717 plus Autosampler, quaternary LC-10A VP pumps, and a programmable 2487 dual λ absorbance UV-visible detector. The separation of LUT was carried out using a Nucleodur (150 mm × 4.6 mm) RP C_8_ column filled with 5 μm filler as a static phase. The binary mixture of 0.1 % formic acid and acetonitrile (7:3 % *v/v*) was used as the mobile phase. The mobile phase was flowed with a flow rate of 1.0 mL/min and detection was performed at 348 nm. The injection volume for analytes was set at 20 μL. Millennium (version 32) software was used for the data analysis.

### 3.3. Solubility Study of LUT in Different Components

Based on LUT’s equilibrium solubility data, various components were chosen. Solubility studies were performed in order to select suitable components for the preparation of LUT SNEDDS instead of optimizing LUT dose. Using a saturation shake flask method, the equilibrium solubility of LUT in various oils (Lauroglycol-90, Lauroglycol-FCC, Capryol-90, Capryol-PGMC, Triacetin, and sesame oil), surfactants (Labrasol, Tween-80, and Cremophor-EL), co-emulsifiers (Transcutol-HP, EG, PG, ethanol, and IPA), and water was estimated [58]. These experiments were carried out in triplicate with an excess amount of LUT mixed in with known proportions of various components. After being vortexed for approximately 5 min, the samples were then transferred to a WiseBath^®^ WSB Shaking Water Bath (Model WSB-18/30/-45, Daihan Scientific Co. Ltd., Seoul, Korea) for continuous shaking. These tests were carried out at a temperature of 25 ± 0.5 °C; 100 rpm and 72 h were chosen as the speed and equilibrium time, respectively. The samples were removed from the shaker and centrifuged at 5000× *g* rpm once equilibrium was reached. The supernatants were carefully removed, diluted (as needed) with the mobile phase, and utilized to analyze LUT using the HPLC technique at 348 nm [23].

### 3.4. Construction of Pseudo-Ternary Phase Diagrams for Preparation of SNEDDS

Capryol-PGMC (oil), Tween-80 (surfactant), and Transcutol-HP (co-emulsifier) were chosen for the manufacture of LUT SNEDDS based on the equilibrium solubility data of LUT in various oils, surfactants, and co-emulsifiers. Because water is frequently utilized in the literature [42,43,44], it was chosen as the aqueous phase. Low-energy emulsification was used to create phase diagrams [35,43]. As a result, the surfactant (Tween-80) and co-emulsifier (Transcutol-HP) were properly blended in different mass ratios, such as 1:0, 1:2, 1:1, 2:1, 3:1, and 4:1 mass ratios. The oil phase, i.e., Capryol-PGMC, was combined with a specified S_mix_ ranging from 1:9 to 9:1. By slowly adding water, the oil phase and certain S_mix_ mixes were titrated, and visual observations were made based on their transparency/clarity. Upon each addition of water, the physical appearance was recorded. The formulations showing clear/transparent and easily flowable behavior were selected, and other formulations such as turbid emulsions, turbid gels, and translucent gels were discarded based on visual observations. At this stage, visual observations were made. However, selected formulations were fully characterized for thermodynamic stability, self-nanoemulsification efficiency, and various physicochemical parameters, which are detailed in the next sections. For each S_mix_ ratio, the SNEDDS zones were built individually on pseudo-ternary phase diagrams [28,44], where one axis representing oil phase, second representing aqueous phase, and third representing a specific S_mix_ ratio (Figure 1). 

The highest SNEDDS zones were represented by a 1:1 mass ratio of Tween-80 and Transcutol-HP, according to phase diagrams. As a result, utilizing a 1:1 S_mix_ ratio, multiple SNEDDS formulations for LUT were chosen. Different SNEDDS with assigned codes of LSN1–LSN5 were accurately picked from a 1:1 S_mix_ ratio. In the phase diagram, the complete region of SNEDDS zones was considered. Different concentrations of Capryol-PGMC (12, 16, 20, 24, and 28 percent *w/w*) with consistent amounts of Tween-80 (20 percent *w/w*) and Transcutol-HP (20 percent *w/w*) were selected from the phase diagram for the manufacture of LUT SNEDDS. In each SNEDDS, 20 mg of LUT was included.

### 3.5. Thermodynamic Stability Tests

LUT SNEDDS (LSN1-LSN5) underwent various thermodynamic stability tests in order to eliminate metastable/unstable SNEDDS. Centrifugation, heating and cooling cycles, and freeze-pump thaw cycles were used in these tests [28,35]. For approximately 30 min, LUT SNEDDS were centrifuged at 5000× *g* rpm and observed for any physical changes. SNEDDS that were centrifugally stable were exposed to further heating and cooling cycles. Four heating and cooling cycles were carried out at temperatures ranging from 4 to 45 °C for a total of 48 h at each temperature. Freeze-pump-thaw cycles were used on SNEDDS that were stable during the heating and cooling cycles. Four freeze-pump-thaw cycles were carried out at temperatures ranging from −21° to 25 °C for a total of 24 h at each temperature. Finally, those SNEDDS that passed all three steps of thermodynamic stability tests were chosen for future study.

### 3.6. Self-Nanoemulsification Test

Using an A–E grading systems, a self-nanoemulsification test was performed to investigate any drug precipitation or phase separation after dilution with various diluents [28,35]. For this test, three different diluents were used: deionized water, 0.1N HCl, and phosphate buffer (pH 6.8). Each SNEDDS (LSN1-LSN5) had its self-nanoemulsification efficiency assessed visually using the A–E grading systems, as reported previously [28,35]. Only those SNEDDS who received an A or B on this test were chosen for further evaluation.

### 3.7. Physicochemical Characterization of SNEDDS

Droplet size distribution, PDI, ZP, surface morphology, RI, and percent T were all measured in LUT SNEDDS (LSN1-LSN5). A Malvern Zetasizer (Nano ZS90, Malvern Instruments Ltd., Holtsville, NY, USA) was used to determine the droplet size and PDI of the LUT SNEDDS. The experiments were conducted at a temperature of 25 °C and a scattering angle of 90°. Approximately 1 mL of each LUT SNEDDS was diluted with water (1:200) and 3 mL of each diluted SNEDDS was transferred to an acrylic plastic cuvette to determine droplet size and PDI. A Malvern Zetasizer (Nano ZS, Malvern Instruments Ltd., Holtsville, NY, USA) was used to determine the ZP of each LUT SNEDDS. The process for determining ZP was the same as that for determining droplet size and PDI, with the exception that samples were taken into glass electrodes for ZP measurement.

An Abbes type Refractometer (Precision Standard Testing Equipment Corporation, Darmstadt, Germany) was used to determine the RI of each LUT SNEDDS. Castor oil was utilized as the standard, and RI measurements were performed on undiluted samples. The turbidity/percent T of each LUT SNEDDS was determined using a UV-Visible Spectrophotometer (SP1900, Axiom, Germany) at 550 nm according to the literature [28]. 

Transmission Electron Microscopy (TEM) was used to investigate the surface morphology and form of an optimized SNEDDS (LSN1). JEOL TEM (JEOL JEM 2100 F, USA) was used to conduct TEM on LSN1. Central Laboratory, Research Center, College of Science, King Saud University, Riyadh, Saudi Arabia conducted the TEM evaluation. Optimized SNEDDS LSN1 was diluted as stated for droplet size and PDI assessment. On a carbon-coated grid, a drop of diluted SNEDDS LSN1 was placed and left to dry. The experiment was carried out using a TEM at 80 KV.

### 3.8. In Vitro Drug Release Evaluation 

Using a dialysis bag (MWCO: 12-14 KDa; Spectrum Medical Industries, Mumbai, India), in vitro drug release studies of LUT from five distinct SNEDDS (LSN1-LSN5) were carried out and compared to a control (LUT suspension) [28]. These tests were carried out using 200 mL of phosphate buffer (pH 6.8) as a dissolution medium. The dissolution media was filled in suitable glass beakers. Approximately 1.0 mL of each formulation of LUT and LUT suspension (each formulation containing 20 mg of LUT) were transferred to a dialysis bag, which was clamped using plastic clips. The dialysis bags clamped with plastic clips were immersed into glass beakers containing 200 mL of dissolution media. The whole assembly was placed into a WiseBath^®^ WSB Shaking Water Bath (Model WSB-18/30/-45, Daihan Scientific Co. Ltd., Seoul, Korea) at 37 ± 1.0 °C for shaking at 100 rpm. At different time periods, 1.0 mL of samples from each formulation were carefully withdrawn and replaced with the same volume of freshly made LUT free dissolution media. The concentration of LUT in each SNEDDS and LUT suspension was measured using the HPLC technique at 348 nm at each time interval [23].

### 3.9. Hepatoprotective Effects

Thirty male Wistar Albino rats weighing 200–250 mg/kg were donated by the Experimental Animal Care Center (EACC) at Prince Sattam bin Abdulaziz University, Al-Kharj, Saudi Arabia. Before the experiment began, all the animals were acclimatized and kept in plastic cages in typical laboratory settings for animal care and storage, and they were fed a regular pellet diet with water ad libitum. The EACC, Prince Sattam bin Abdulaziz University, Al-Kharj, Saudi Arabia provided the recommendations for all experimental protocols and procedures. The Animal Ethics Committee of the EACC Board (Prince Sattam bin Abdulaziz University, Al-Kharj, Saudi Arabia with approval number: BERC-017-10-21) gave its approval to these investigations. Animals were used, and experimental techniques followed the European Union (EU) directive 2010/63/EU.

Because CCl_4_ has been identified as a suitable toxicant for hepatotoxicity [59], it was used to induce hepatotoxicity. The rats were put into five groups at random, with six rats in each group. For 5 days, Group I animals were given a daily dose of 1 mL aqueous solution of 0.5 percent w/w carboxymethyl cellulose (CMC) (p.o.) as a control. As a toxic control, Group II animals were given a daily dosage of aqueous solution containing 0.5 percent *w/w* CMC (p.o.) and a single dose of CCl_4_ (1 mg/kg, i.p.) on day 1. The standard group consisted of animals that were given an oral suspension of standard silymarin (10 mg/kg) on all 5 days and CCl_4_ (1 mg/kg, i.p.) on days 2 and 3 following 1 h of silymarin administration. The test LUT group consisted of animals that were given LUT suspension (20 mg/kg) for all 5 days and CCl_4_ (1 mg/kg, i.p.) on days 2 and 3 following 1 h of LUT suspension administration. The test LUT SNEDDS LSN1 group consisted of rats that were given optimized LUT SNEDDS LSN1 (containing 20 mg/kg of LUT) on all 5 days and CCl_4_ (1 mg/kg, i.p.) on days 2 and 3 after 1 h of receiving LUT SNEDDS LSN1. All animals were starved overnight on day 6, and 1.5–2.0 mL of blood was collected in sterile Eppendorf tubes from the tail vein and maintained at 37 °C for around 45 min. After centrifugation at 3000 rpm for 15 min, the serum was separated using a sterile micropipette. In order to assess liver function, serum samples were subjected to biochemical analysis. Various biochemical markers, such as serum AST, serum ALT, serum ALP, serum γ-GGT, and serum bilirubin were calculated following protocols described in the literature [60,61,62].

### 3.10. Estimation of Biomarkers of Liver Tissue

Fresh livers from rats were obtained and weighed precisely. In 1.5 M KCl, liver homogenates were produced at 10 percent *w/v*. In liver homogenates, biochemical markers such as CAT, GSH, SOD, and MDA were measured, as reported in the literature [63,64].

### 3.11. Statistical Evaluation

In vitro experiment results are presented as the mean ± SD of three independent experiments. The results of hepatoprotective studies, on the other hand, are given as the mean ± SD of six independent experiments. All the results were statistically assessed by one way analysis of variance (ANOVA) followed by Dennett’s test using GraphpadInstat software (San Diego, CA, USA). The significant value was defined as the *p* value at the 5% level of significance (*p* < 0.05).

## 4. Conclusions

Hepatoprotective effects of a weakly soluble bioactive flavonoid LUT were evaluated using SNEDDS formulations. Different SNEDDS formulations of LUT were created using a low energy emulsification approach, characterized physicochemically, and tested for in vitro drug release utilizing a dialysis bag. The hepatoprotective effects of SNEDDS formulation LSN1 in rats were explored further based on the minimal Z-average value, maximum drug release profile, and the existence of a minimum concentration of Capryol-PGMC. When compared to LUT suspension and other SNEDDS examined, optimized SNEDDS LSN1 revealed a considerable in vitro drug release profile of LUT. Furthermore, as compared to LUT suspension, the hepatoprotective effects of optimized SNEDDS LSN1 were found to be considerable. Overall, the findings of this study revealed that SNEDDS has the potential to improve LUT’s in vitro dissolution rate and hepatoprotective effects. 

## Figures and Tables

**Table 1:** Equilibrium solubility data of luteolin (LUT) in different excipients at 25 °C.

Components	Equilibrium Solubility (mg/g) *
Triacetin	3.22 ± 0.18
Lauroglycol-90	11.48 ± 1.10
Lauroglycol-FCC	10.79 ± 0.74
Capryol-90	22.42 ± 1.41
Capryol-PGMC	25.72 ± 1.74
Sesame oil	1.58 ± 0.02
Labrasol	14.24 ± 0.59
Tween 80	18.52 ± 0.81
Cremophor-EL	16.83 ± 0.94
EG	6.07 ± 0.28
PG	8.24 ± 0.48
Transcutol-HP	68.32 ± 2.83
Ethanol	11.84 ± 0.87
IPA	12.13 ± 1.08
Water	0.03 ± 0.00

* Values are presented as mean ± SD (*n* = 3).

**Table 2:** Composition of self-nanoemulsifying drug delivery system (SNEDDS) prepared using Capryol-PGMC, Tween-80, Transcutol-HP, and deionized water.

Codes	SNEDDS Components (% *w/w*)					S_mix_ Ratio
	LUT (mg)	Capryol-PGMC	Tween-80	Transcutol-HP	Water	
LSN1	20	12.00	20.00	20.00	48.0	1:1
LSN2	20	16.00	20.00	20.00	44.0	1:1
LSN3	20	20.00	20.0	20.0	40.0	1:1
LSN4	20	24.00	20.0	20.0	36.0	1:1
LSN5	20	28.0	20.0	20.0	32.0	1:1

**Table 3:** Qualitative results of thermodynamic stability and self-nanoemulsification efficiency of LUT-SNEDDS in the presence of different diluents.

SNEDDS	* Test Grade	Thermodynamic Stability Tests		
		C/F	H/C Cycles	F/T Cycles
LSN1	A	✓	✓	✓
LSN2	A	✓	✓	✓
LSN3	A	✓	✓	✓
LSN4	A	✓	✓	✓
LSN5	A	✓	✓	✓

* All the formulations passed this test with Grade-A in the presence of deionized water, 0.1 N HCl, and phosphate buffer (pH 6.8); ✓ (passed the test); C/F (centrifugation); H/C (heating and cooling); F/T (freeze-pump-thaw).

**Table 4:** Physicochemical parameters for various LUT-SNEDDS (mean ± SD, *n* = 3).

Formulations	Characterization Parameters				
	*Z*-Average ± SD (nm)	PDI	ZP ± SD (mV)	RI ± SD	% T ± SD
LSN1	48.58 ± 2.47	0.168	−30.58 ± 1.64	1.344 ± 0.01	98.94 ± 0.53
LSN2	67.25 ± 5.08	0.194	−28.27 ± 1.49	1.347 ± 0.04	98.68 ± 0.28
LSN3	85.84 ± 6.89	0.254	−26.29 ± 1.24	1.348 ± 0.09	97.28 ± 0.25
LSN4	102.58 ± 8.64	0.284	−24.84 ± 1.38	1.349 ± 0.02	95.02 ± 1.24
LSN5	124.58 ± 9.41	0.293	−23.74 ± 2.14	1.345 ± 0.07	94.27 ± 1.09

**Table 5:** Correlation coefficients and kinetics of drug release from SNEDDS (LSN1-LSN) and LUT suspension.

Formulation	Zero Order		First Order		Higuchi	Hixon-Crowell	Peppas	
	K_0_	R^2^	k_1_	R^2^	R^2^	R^2^	R^2^	n
LSN1	11.74	0.913	1.82	0.969	0.968	0.959	0.991	0.981
LSN2	10.88	0.942	1.62	0.982	0.981	0.982	0.992	0.978
LSN3	9.50	0.972	1.44	0.986	0.988	0.983	0.990	0.980
LSN4	7.57	0.980	1.28	0.984	0.983	0.982	0.993	0.992
LSN5	6.34	0.981	1.21	0.984	0.970	0.985	0.992	1.201
LUT suspension	3.13	0.977	1.08	0.985	0.902	0.969	0.993	1.368

Correlation coefficient (R^2^), Zero order rate constant (K_0_), first order rate constant (k_1_), diffusion coefficient (n).

**Table 6:** Influence of LUT-SNEDDS (LSN1) and LUT suspension administrations on different biomarkers of rat serum.

Groups	AST (U/L)	ALT (U/L)	ALP (U/L)	γ-GGT (U/L)	Bilirubin (U/L)
I	76.48 ± 1.89	34.69 ± 0.96	99.58 ± 2.65	1.50 ± 0.06	0.75 ± 0.02
II	224.41 ± 5.89	96.61 ± 1.96	227.45 ± 6.14	3.74 ± 0.12	1.10 ± 0.03
III	95.21 ± 0.98	45.24 ± 1.78	110.58 ± 1.95	1.97 ± 0.02	0.73 ± 0.02
IV	175.28 ± 4.57	68.29 ± 1.89	158.68 ± 3.59	2.58 ± 0.05	0.90 ± 0.04
V	102.24 ± 2.64	51.28 ± 1.38	112.12 ± 2.52	1.89 ± 0.07	0.70 ± 0.01
Normal levels	75.80 ± 1.04	33.94 ± 0.98	81.09 ± 1.80	1.26 ± 0.06	0.72 ± 0.01

**Table 7:** Influence of LUT-SNEDDS (LSN1) and LUT administrations on different biomarkers of rat liver.

Groups	CAT (U/mg)	GSH (nmol/mg)	MDA (nmol/mg)	SOD (U/mg)
I	47.71 ± 1.29	1.19 ± 0.03	3.25 ± 0.07	24.86 ± 1.34
II	18.14 ± 2.98	0.45 ± 0.01	11.51 ± 0.41	9.81 ± 0.45
III	44.41 ± 1.87	0.98 ± 0.02	3.98 ± 0.28	20.16 ± 0.81
IV	29.81 ± 1.18	0.75 ± 0.01	6.14 ± 0.17	17.12 ± 0.91
V	41.14 ± 0.91	0.96 ± 0.03	3.87 ± 0.16	19.15 ± 0.37
Normal levels	45.09 ± 1.07	1.17 ± 0.02	3.20 ± 0.10	22.24 ± 0.41

## Data Availability

This study did not report any data.

## Sample Availability

Samples of the LUT compounds are available from the authors.
